# The effect of an open‐heart surgery patient care protocol on post‐sternotomy pain, anxiety and quality of care: A randomized controlled trial

**DOI:** 10.1111/nicc.13193

**Published:** 2024-10-16

**Authors:** Necibe Dağcan Şahin, Gülşah Gürol Arslan

**Affiliations:** ^1^ Faculty of Health Sciences Kutahya Health Sciences University Kutahya Turkey; ^2^ Fundamentals of Nursing Department, Nursing Faculty Dokuz Eylül University Izmir Turkey

**Keywords:** anxiety, care protocol, open‐heart surgery, pain, quality of care

## Abstract

**Background:**

Open‐heart surgery patients face many problems because of post‐sternotomy pain. Care protocols can eliminate pain and pain‐related problems by providing holistic care.

**Aim:**

The aim of this study was to examine the effect of an open‐heart surgery patient care protocol developed in the study on post‐sternotomy pain, anxiety and quality of care.

**Study Design:**

The study was carried out as a double‐blind randomized controlled trial. The sample size was calculated. Considering some attrition, the sample size was increased by 10% for each group, and a total of 68 participants, including 34 in each group, were included in the sample. Data were collected using a ‘Patient Information Form’, a ‘Post‐Sternotomy Pain Follow‐up Form’, the ‘Numeric Rating Scale’, the ‘State Anxiety Inventory’ and the ‘Strategic and Clinical Quality Indicators in Postoperative Pain Management Questionnaire’. The patients in the experimental group were given care in accordance with the protocol, which was developed in the study, on postoperative days 0, 1 and 2.

**Results:**

The statistical evaluation showed a significant difference between the mean scores of the experimental (*F* = 7.28; *p* < .001) and control groups (*F* = 2.42; *p* < .05) on the pain assessment scale. It was determined that the number of analgesics used in the experimental group was statistically significantly lower than in the control group. Intra‐group comparisons showed that there was a difference between the mean pre‐test and post‐test state anxiety scale scores of the groups (*p* < .001). The experimental group had higher mean scores on the Strategic and Clinical Quality Indicators in Postoperative Pain Management Questionnaire than that of the control group (*p* < .001).

**Conclusions:**

The protocol developed in the study was found to be effective in reducing pain, the use of NSAIDs and opioids, and anxiety levels and increasing the level of quality of care.

**Relevance to Clinical Practice:**

The protocol was original and feasible in that it included independent nursing interventions to improve the quality of care by reducing pain and anxiety. Particularly, the use of protocols in intensive care units was nurses' strongest resource in patient care management. Thus, the protocol, which was prepared for intensive care patients who most frequently experience pain and anxiety, was promising for nurses in improving the quality of care by reducing pain and anxiety. However, it is necessary to conduct further studies involving longitudinal follow‐up in samples and institutions with similar conditions.

**What is known about the topic**

- After open‐heart surgery, patients experience pain, and the pain affects anxiety and quality of care.
- Patients should be treated holistically after surgery. Protocols should be preferred to provide holistic care.

**What this paper adds**

- This study provided an overview of patients' pain, anxiety and quality of care satisfaction after heart surgery.
- The protocol showed promise in improving quality of care by reducing pain and anxiety.

## 1 INTRODUCTION

Although developments in health technology are used to prevent most diseases, cardiovascular diseases continue to threaten human life.^1^ According to the World Health Organization (WHO), coronary artery disease (CAD) is among the top 10 causes of death.^2^ Some sternotomy‐related symptoms are observed in the open‐heart surgery method used for the treatment of cardiovascular diseases. Patients' daily living activities are affected following surgery, and patients are exposed to internal and external threats.^3^ Patients undergoing cardiac surgery are followed in intensive care, and it has been reported that pain and anxiety are the main stress factors of this care.^1^, ^4^

## 2 BACKGROUND

Sternotomy is performed on more than 2 million people globally every year, and individuals experience constant pain in the anterior thorax.^5^, ^6^ Despite gradually developing pain management and treatment methods, pain is the main complaint in individuals undergoing cardiovascular surgery, and they complain of moderate to severe pain even at rest.^5^, ^7^ Uncontrolled pain causes physical and psychological problems in individuals, prolongs hospital stays and increases costs.^8^, ^9^ International guidelines recommend using a protocol‐based, stepwise approach to pain management of critically sick adults.^10^, ^11^ In addition, the American Pain Society (APS) recommends that the institutions where the surgery will take place have an organizational structure that can improve policies and processes for safe and effective postoperative pain control.^12^

Another complication that occurs after open‐heart surgery is anxiety.^13^ Preoperative and postoperative anxiety in patients undergoing cardiac surgery disrupts coronary circulation and increases the risk of myocardial infarction.^14^ Even indirectly, anxiety increases the pain experienced after cardiac surgery, causing the use of excessive amounts of analgesics.^15^ In addition, following patients in intensive care after cardiac surgery disrupts the physical and psychological conditions of those experiencing pain and causes their anxiety levels to increase. Factors, such as being in intensive care, being away from home and work, lack of social support and long‐term treatment, cause anxiety in patients.^14^, ^15^ Nurses have a great responsibility for managing the anxiety of patients undergoing coronary artery bypass graft (CABG) surgery. Sedative medications may often be preferred in the management of anxiety. However, it has been reported that such drugs cause respiratory depression.^14^ In addition, nurses can reduce the anxiety level by providing pain control in the anxiety management of patients undergoing cardiac surgery.^16^ In addition, some studies have shown that nurses play a role in the management of patients' anxiety process by using nursing interventions such as music,^17^ affirmation statements,^13^ aromatherapy,^18^ information about the duration of surgery, counselling^19^ and education.^20^

Nursing care quality is the complete meeting of patients' needs during the medical diagnosis and treatment process by nurses.^21^ One of the most critical indicators of the quality of care is the level of patients' perceptions of care. For this reason, it is important to measure the quality of patients' care and satisfaction levels to evaluate the quality of care.^22^ Quality care also increases the quality of health services. Evidence‐based and individualized care applied by nurses will become stronger with protocols and will have a significant place in increasing quality.^23^, ^24^

The guidelines emphasize that processes using a protocol‐based, step‐by‐step approach are important in symptom management of critically ill patients. In this context, the effectiveness of the care protocol developed in our study was assessed with the pain, anxiety and quality of care results of cardiovascular surgery intensive care patients.

## 3 AIM

It is thought that a care protocol will be useful in addressing pain, anxiety and quality of care holistically for patients undergoing open‐heart surgery. No studies have been found in the literature on the examination of the effects of nursing care protocols on pain, anxiety and quality of care in patients undergoing open‐heart surgery. For this reason, this study was conducted to develop a patient care protocol for intensive care patients undergoing open‐heart surgery and to examine the effect of the protocol on post‐sternotomy pain, anxiety and quality of care. It is thought that the study will shed light on the literature by serving as a resource in the management of pain, anxiety and quality of care. In addition, this protocol is expected to provide standardization and improve results by preventing differences between post‐sternotomy patient care practices.

### 3.1 Hypotheses

The open‐heart surgery patient care protocol reduces pain.

The open‐heart surgery patient care protocol reduces the patient's anxiety level.

The open‐heart surgery patient care protocol improves the quality of patient care.

## 4 DESIGN AND METHODS

The study was completed as a double‐blind randomized controlled trial. This study was conducted, and results were reported in accordance with the CONSORT guideline.^25^

### 4.1 Setting and sample

The research included patients who underwent open‐heart surgery and were followed in the cardiovascular surgery intensive care unit following surgery. The sample size was calculated on the G*Power 3.1.9.7 software considering independent groups *t*‐test. The results of the study by Chandrababu et al.^26^ were taken as a reference in the calculation of the effect size used in the study.^26^ The minimum sample size for each group was calculated as 31 individuals by considering an effect size of *d* = 0.72, a margin of error of 5% (*α* = 0.05) and a power value of 80% (1 − *β* = 0.80) in the power analysis which was conducted using the mean pain scores in the study by Chandrababu et al.^26^ Considering some attrition, the sample size was increased by 10% for each group, and a total of 68 participants, including 34 in each group, were included in the sample.^27^, ^28^

### 4.2 Inclusion criteria

Patients who agreed to participate in the study, underwent CABG surgery, had sternotomy, were aged >18 years, were cognitively competent, were extubated and were conscious and oriented were included in the study.

### 4.3 Exclusion criteria

Patients who wanted to quit during the study, developed delirium, had to undergo emergency surgery, used anxiolytics, had alcohol and drug addiction, had chronic psychiatric and pain problems, developed complications (inability to discontinue mechanical ventilator support, inability to discontinue inotropic support) until post‐operative transfer to the clinic or had intra‐aortic balloon pumps or pacemakers were not included in the study.

### 4.4 Randomization and blinding

**FIGURE 1:**
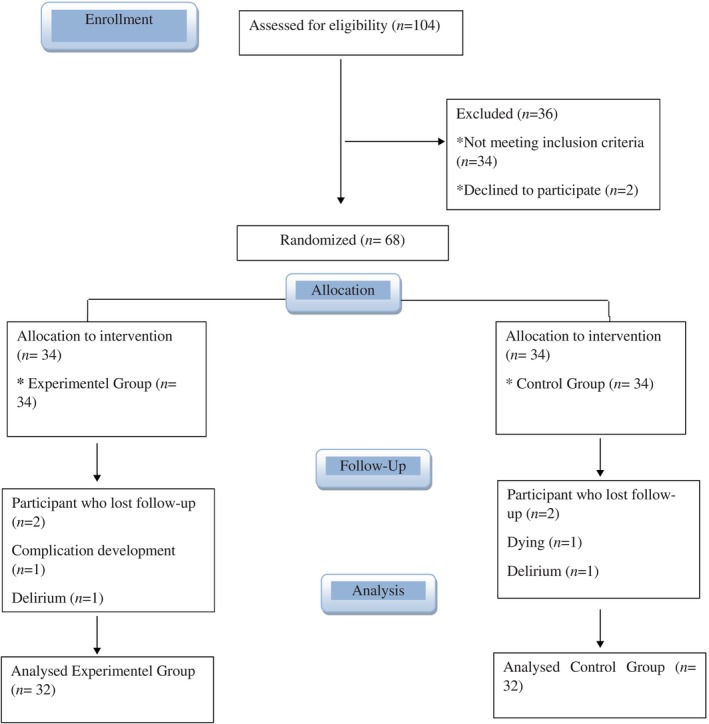
CONSORT flow diagram.

The patient group to which the open‐heart surgery patient care protocol was applied was named the experimental group, and the group to which the routine nursing care of the intensive care unit was applied was named the control group. Sixty‐eight patients selected via the consecutive sampling method were assigned to the experimental and control groups by block randomization method using random number generation software (Research Randomizer). Two sets of 34 unique numbers in the range of 1–68 were created on the software, and patients were randomly assigned to experimental and control groups. Experimental and control group applications were carried out by nurses. Data were collected by the first researcher. The groups were labelled as Group 1 and Group 2 in the statistical analysis of the data, and the researcher was blinded, while the results were evaluated. Therefore, researchers did not know which group was the experimental group and which group was the control group. To reduce the interaction between the control and experimental groups, patients were placed in different areas in the intensive care unit, which consisted of two sections, and thus, the patients were blinded, too. Figure 1 shows the enrolment, allocation, follow‐up and analysis stages by using CONSORT.

At the end of the study, a “post hoc” power analysis was performed using the mean pain scores of experimental and control groups on the G‐Power statistical software following the application. The effect value of our study was calculated as 2.54, and post hoc power analysis was performed at *ἀ* = 0.05. The power of our study was determined as 99%. The study was completed with 64 patients (32 in the experimental and 32 in the control group).

### 4.5 Data collection tools

The primary main outcomes of our study were pain, anxiety and quality of care score. Data were collected with a Patient Information Form, which was created by the researchers; a Post‐Sternotomy Pain Follow‐up Form; the Numeric Rating Scale, the State Anxiety Scale and the Strategic and Clinical Quality Indicators in Postoperative Pain Management Questionnaire.

#### 4.5.1 Patient information form

This form consists of a total of five questions about demographic information, such as the patient's age, gender, educational status, previous surgical intervention and comorbidity status.

#### 4.5.2 Post‐sternotomy pain follow‐up form

This form consists of three questions about the patient's pain‐related information, such as severity of pain and type and number of analgesics.

#### 4.5.3 The numeric rating scale (NRS)

Pain is rated from 0 to 10 on this scale. A score of 0 represents no pain at all, and a score of 10 represents the worst pain possible. The scale, whose validity and reliability has been established, is widely used because it facilitates the identification of pain.^29^, ^30^

#### 4.5.4 The state–trait anxiety inventory (STAI)

This scale was developed by Spielberger in 1964.^31^ Its Turkish adaptation was conducted in 1974–1977.^32^ Cronbach's alpha coefficient was found to be between .94 and .96. The scale consists of a total of 20 items, and each item is evaluated with the following options: (1) not at all, (2) a little, (3) a lot and (4) completely. One point shows low anxiety and four points high anxiety. Positive emotions are defined with reversed statements, and the scores are also reversed; that is, one point is calculated as four, and four is calculated as one. The total score is between 20 and 80. According to the scale scores, anxiety is categorized as none (0–19), mild (20–39), moderate (40–59) and high (60–80).

#### 4.5.5 The strategic and clinical quality indicators in postoperative pain management questionnaire

This scale was developed by Idval et al.,^33^ and its Turkish validity and reliability study was performed by Vatansever and Akansel.^34^ The scale consists of 14 items. In addition to these 14 items, there are five more questions, two of which are used to evaluate general patient satisfaction and three to evaluate pain severity. Because these variables were evaluated by using a separate form in our study, this section was not used. Items are scored on a Likert‐type scale with options from ‘strongly disagree’ (1 point) to ‘strongly agree’ (5 points). The lowest score is 14, and the highest score is 70. A high score indicates that the patient perceives the quality of postoperative care as high. It also shows that the patient's satisfaction is high. Cronbach's alpha value of the scale is 0.81.

### 4.6 Interventions

The study consisted of two stages. The first stage is the development of the Open‐Heart Surgery Patient Care Protocol. The second stage is the implementation and evaluation of the protocol developed.

#### 4.6.1 Stage one: development of the protocol

Following a review of the literature and international guidelines, a draft care protocol was prepared by the researchers.^4^, ^8^, ^10^, ^12^, ^24^, ^35^, ^36^, ^37^ The number of personnel in the unit and institutional conditions were taken into consideration during the development of the protocol. Patients in the intensive care unit where the research was conducted are followed up on postoperative days 0, 1 and 2 following CABG surgery, and if no complications develop, they are transferred to the clinic. Accordingly, the protocol was planned to include post‐operative days 0, 1 and 2. In the protocol, a line of variance codes was created for nurses to add positive and negative variances under each main factor, and the variance codes were determined as A (problems originating from the patient), B (problems originating from the health care team) and C (problems originating from the institution). Before the protocol was implemented, five experts in the field (one cardiovascular surgery nurse; three nurse academicians specialized in nursing principles, surgical nursing and pain management; and one cardiovascular surgery specialist physician) were consulted about the ‘Open‐Heart Surgery Patient Care Protocol’. The experts were asked to evaluate each item with ‘not appropriate = 1’, ‘somewhat appropriate = 2 (items/phrases need to be changed)’, ‘quite appropriate = 3’ and ‘very appropriate = 4’ and write their suggestions under the items. In line with the expert recommendations, the content validity ratio (CVR) was calculated using the Davis technique. The content validity index (CVI) was calculated for each item based on expert opinions, and items with a CVI value of <0.80 were removed from the protocol. Experts were also consulted about the clarity, appropriateness and language of the protocol items, and necessary corrections were made.^38^ To test the clarity of the draft protocol, a pilot application was performed with five patients who met the inclusion and exclusion criteria. Data obtained from the pilot application were not included in the study. As a result of expert opinions and preliminary evaluations, the items of the protocol were revised and then finalized.

The open‐heart surgery patient care protocol consisted of the following interventions: patient delivery/visits, tests, patient evaluation/diagnostic procedures, medication administration, pain management, treatment/clinical procedures, patient care/nursing interventions, psycho‐social support, hydration/nutrition, activity/environmental safety, providing the patient with information, service transfer plan and evaluation. The protocol, which was prepared in line with the literature, the patient group and institutional conditions, was implemented by experienced and expert nurses within a timetable.

The existing nursing care protocol of the intensive care unit included interventions, such as patient delivery, treatment practices, provision of nursing care and implementation of interventions, and transfer from intensive care. The existing nursing care content in the institution was carried out in line with the nurse's work intensity, the patient's needs and the physician's request.

#### 4.6.2 Stage two: implementation and evaluation of the protocol

In the first step, eight nurses who volunteered to participate in the study had at least 4 years of working experience in the cardiovascular surgery intensive care unit, and could actively work shifts were selected. The nurses who would provide care for the experimental group were trained with the ‘Open‐Heart Surgery Patient Care Protocol Training Booklet’. The training was conducted in the meeting room of the institution on a day when nurses were not working and lasted approximately 45 min. It was made sure that these nurses provided care only for the experimental group patients. The care of the control group patients was provided by intensive care nurses. The existing nursing care protocol of the intensive care unit was implemented for the control group patients.

In the second step, 1 day before the surgery, the experimental group patients were given approximately 10 min of training with the ‘Open‐Heart Surgery Patient Education Brochure’ developed in line with the literature and international guidelines,^4^, ^8^, ^10^, ^12^, ^24^, ^35^, ^36^, ^37^ and the Patient Information Form was filled out. On the morning of surgery, the patients in the experimental and control groups filled out the state anxiety inventory before they were transferred to the operating room.

In the third step, after the patients in both groups were extubated on postoperative day 0, the ‘Post‐Sternotomy Pain Follow‐up Form’ was filled out. The protocol developed in the study was used to provide care for patients in the experimental group by volunteer nurses. The patients in the control group were provided care according to the existing nursing care protocol of the intensive care unit. Patients' pain levels were measured every 1 h and 15 min after extubation, every 2 h for up to 4 h and every 4 h until transfer to the ward.^39^ Immediately after the patients in both groups were transferred to the ward, they were asked to fill out the state anxiety scale and the strategic and clinical quality indicators in postoperative pain management questionnaire.

## 5 DATA ANALYSIS

The data were analysed by the researcher on the IBM SPSS (Statistical Package for Social Sciences) 29 software with the support of an independent statistician. Normal distribution assumption was checked by ensuring that the skewness and kurtosis values fall within range of −1 to +1. In addition, variance homogeneity was checked using the Levene's test and, sphericity controlled with Mauchly's test. As a result of the analysis, it was found that the data were normally distributed. Categorical data were presented using frequency and percentage values, and scale data were shown with mean and standard deviation values as they met the assumption of normality. When the sample size assumption in the analysis of categorical variables (expected value >5 in at least 20% of the cells) was met, the chi‐square test was applied. When this assumption was not met, Fisher's exact test was used. Paired samples *t*‐test was used for comparing two dependent groups, while the independent samples *t*‐test was used for comparing independent groups. Two‐way repeated measures analysis of variance (Greenhouse–Geisser statistic) test was performed to examine the interaction effect of the differences between three dependent groups. Adjusted Bonferroni tests were used to determine the group or groups that created the difference. The level of significance was taken as *p* < .05.

### 5.1 Ethical consideration

The patients were informed about the research, and their written and verbal consent was obtained. The permission of the institution where the research would be conducted (05.08.2021), the approval of the Non‐Interventional Research Ethics Committee (decision number: 2021/17‐11; date: 02.06.2021) and the written permission of Vatansever and Akansel for the ‘Strategic and Clinical Quality Indicators in Postoperative Pain Management Questionnaire’ used in the research were obtained. The study was registered at Clinical Trials (registration code: NCT05902052).

## 6 RESULTS

The results of the research conducted to determine the effect of the open‐heart surgery patient care protocol on the post‐sternotomy pain, anxiety and quality of care of experimental and control group patients are presented below.

Table 1 shows the results of the analysis regarding the distribution of the demographic characteristics of the patients in the experimental and control groups. The age difference between the groups was not statistically significant (*t* = −0.12; *p* = .89). Age showed a homogeneous distribution between groups. The gender distribution of the individuals in the experimental and control groups was equal, and 53.1% of the individuals in the experimental group and 62.5% of the individuals in the control group were primary school graduates. It was found that 40.6% of the individuals in the experimental group and 50% of those in the control group had previously undergone a surgical procedure. The comorbidity rate in the experimental group was 71.9%, and the rate in the control group was 62.5%. The experimental group patients felt the most pain in the sternum and the least pain in the back. Control group patients stated that they felt the most pain in the sternum area, back, saphenous vein incision area and drain area, respectively. The demographic characteristics of the experimental and control groups did not show a statistically significant difference (*p* > .05). The distribution of individuals was homogeneous according to their demographic characteristics.

**TABLE 1:** Analysis results about the descriptive characteristics of the patients in the experimental and control groups (*n* = 64).

Descriptive characteristics		Group				Homogeneity test				
		Experimental group (*n* = 32)		Control group (*n* = 32)						
Age (mean ± standard deviation)		61.75 ± 10.29		62.06 ± 9.30		*t* = −0.12; *p* = 0.89			%95 C.I.	
		*n*	%	*n*	%	$\chi^{2}$	*p*	OR	Lower	Upper
Gender	Female	16	50.0	16	50.0	0.00	1.00	1.000	0.375	2.664
	Male	16	50.0	16	50.0			‐	‐	‐
Education	Primary school	17	53.1	20	62.5	3.778	.435	0.680	0.251	1.843
	High school	10	31.3	5	15.6	‐	‐	‐	‐	‐
	University	5	15.6	7	21.9	‐	‐	‐	‐	‐
Previous surgical intervention	Yes	13	40.6	16	50.0	0.567	.451	0.684	0.255	1.839
	No	19	59.4	16	50.0	‐	‐	‐	‐	‐
Comorbidity	Yes	23	71.9	20	62.5	0.638	.424	1.533	0.536	4.389
	No	9	28.1	12	37.5	‐	‐	‐	‐	‐
Pain site	Back	6	18.8	10	31.3	0.144	.986	0.508	0.159	1.620
	Sternum incision	10	31.3	10	31.3	‐	‐	‐	‐	‐
	Saphenous vein incision	7	21.9	7	21.9	‐	‐	‐	‐	‐
	Drain site	9	28.1	5	15.6	‐	‐	‐	‐	‐

As seen in Table 2, a statistical difference was detected in both groups according to the mean NRS scores of the experimental and control groups (*F* = 7.28; *p* < .001) (*F* = 2.42; *p* < .05). The examination of the group * time interaction showed that there were differences between the groups in terms of measurement times (*F* = 2.42; *p* < .05). The intra‐group differences in the experimental and control groups were examined. The measurement differences in the control group were higher than in the experimental group.

**TABLE 2:** The intra‐ and inter‐group distribution of mean NRS scores of the experimental and control groups (*n* = 64).

NRS	Group					
	Experimental group (*n* = 32)			Control group (*n* = 32)		
	Mean	Standard deviation	%95 C.I.	Mean	Standard deviation	%95 C.I.
Following extubation	3.06	2.76	2.1–4.02	4.69	2.35	3.88–5.5
15th minute	2.22	2.68	1.29–3.15	4.72	2.32	3.92–5.52
30th minute	2.03	2.35	1.22–2.84	4.44	2.21	3.67–5.21
45th minute	1.87	2.18	1.11–2.63	4.25	2.26	3.47–5.03
1st hour	1.03	2.06	0.32–1.74	4.16	2.52	3.29–5.03
2nd hour	1.75	1.98	1.06–2.44	4.22	2.68	3.29–5.15
4th hour	1.91	2.31	1.11–2.71	4.34	2.67	3.41–5.27
8th hour	1.25	1.93	0.58–1.92	4.94	2.29	4.15–5.73
12th hour	2.06	2.37	1.24–2.88	4.84	2.32	4.04–5.64
16th hour	1.22	1.88	0.57–1.87	4.47	3.10	3.4–5.54
20th hour	.72	1.22	0.3–1.14	5.13	2.74	4.18–6.08
24th hour	.69	1.86	0.05–1.33	4.78	2.74	3.83–5.73
28th hour	.41	1.24	−0.02 to 0.84	4.47	2.93	3.45–5.49
32nd hour	.50	1.27	0.06–0.94	4.19	2.61	3.29–5.09
36th hour	.31	.86	0.01–0.61	3.56	2.58	2.67–4.45
40th hour	.06	.35	−0.06 to 0.18	3.53	2.79	2.56–4.5
Before transfer to the ward	.38	1.10	0–0.76	2.72	2.64	1.81–3.63
Mean pain score	1.26	.80	0.98–1.54	4.32	1.50	3.8–4.84
Time effect	*F* = 7.28; *p* < .001			*F* = 2.42; *p* = .020		
Group effect				*F* = 102.832; *p* < .001		
Group × time interactions				*F* = 2.42; *p* = .015		

Abbreviations: CI, confidence interval; NRS, numeric rating scale.

According to the adjusted Bonferroni tests conducted for the experimental group, statistically significant differences were found between ‘Following extubation’ and the 16th, 20th, 24th, 28th, 32nd, 36th and 40th h, between ‘Before transfer to the ward’ and ‘Before transfer to the ward’ and between the 15th minute and the 40th hour (*p* < .05). It was observed that NRS scores decreased over time. In the control group, statistically significant differences were found between ‘Before transfer to the ward’ and the 8th hour, the 20th hour, and the pain average (*p* < .05). It was observed that NRS scores decreased over time. Statistically significant differences were found between the experimental and control groups at all measurement times (*p* < .05). The measurements of the control group were higher than those of the experimental group.

**FIGURE 2:**
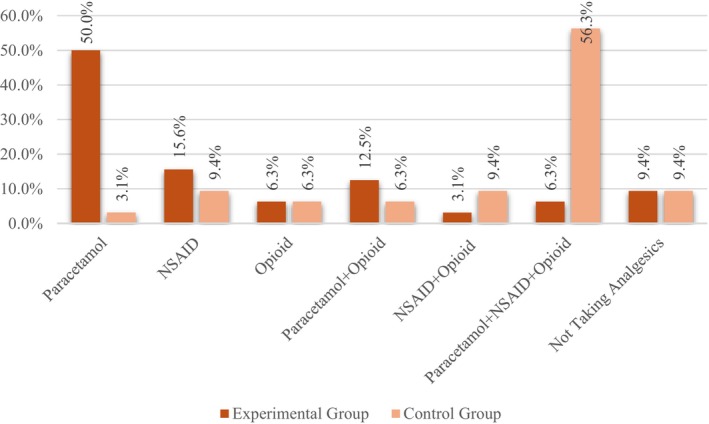
Distribution of experimental and control groups by analgesic use.

The number of analgesics used showed a statistical significance between the groups (*t* = −5.22; *p* < .001). It was higher in the control group (3.22 ± 1.79) than in the experimental group (1.41 ± .80). Figure 2 shows the distribution of the groups according to the number and type of analgesics used. A significant difference was found between the groups in terms of the number of paracetamol use (*X* ^2^ = 18.02; *p* < .001). While the rate of paracetamol use in the experimental group was 50%, it was 3.1% in the control group. A significant difference was found between the groups in terms of the number of paracetamol + NSAID + opioid use (*X* ^2^ = 18.61; *p* < .001). While this rate was 6.3% in the experimental group, it was 56.3% in the control group.

The mean pre‐test and post‐test State Anxiety Scale scores of the individuals in the experimental group were 39.28 ± 9.77 and 28.28 ± 5.25, respectively. These scores were 58.03 ± 7.07 and 44.12 ± 14.61 in the control group, respectively. The difference between mean pre‐ and post‐test scores was found to be statistically significant in both groups (*t* = 6.12; *p* < .001) (Table 3).

**TABLE 3:** The intra‐ and inter‐group distributions of pre‐test and post‐test scores of experimental and control groups on the State Anxiety Scale (*n* = 64).

	Experimental group (*n* = 32)			Control group (*n* = 32)		
	Mean	Standard deviation	%95 CI	Mean	Standard deviation	%95 CI
State Anxiety Scale (pre‐test)	39.28	9.77	35.89–42.66	58.03	7.07	55.58–60.47
State Anxiety Scale (post‐test	28.28	5.25	26.45–30.09	44.12	14.61	39.05–49.18
Paired samples *t*‐test	*t* = 6.12; *p* < .001			*t* = 4.87; *p* < .001		

Abbreviations: CI, confidence interval; *t* = paired samples *t*‐test.

As seen in Table 4, a statistically significant difference was detected between the mean scores of the experimental and control group participants on the Strategic and Clinical Quality Indicators in Postoperative Pain Management Questionnaire (*t* = 12.98; *p* < .001). The mean and standard deviation values were 66.50 ± 4.60 in the experimental group and 44.03 ± 8.63 in the control group. Experimental group patients reported significantly higher levels of quality of care and satisfaction than the patients in the control group.

**TABLE 4:** The intra‐ and inter‐group distributions of the mean Strategic and Clinical Quality Indicators in Postoperative Pain Management Questionnaire scores of the experimental and control groups (*n* = 64).

	Experimental group (*n* = 32)		Control group (*n* = 32)		t	p
	Mean ± standard deviation	%95 C.I.	Mean ± standard deviation	%95 C.I.		
Strategic and Clinical Quality Indicators in Postoperative Pain Management Questionnaire	66.5 ± 4.6	64.91–68.10	44.03 ± 8.63	41.04–47.02	12.98	**<0.001**

Abbreviations: CI, confidence interval; *t*, independent samples *t*‐test.

## 7 DISCUSSION

In this study, which was conducted to develop an open‐heart surgery patient care protocol and examine its effect on post‐sternotomy pain, anxiety and quality of care, the patients in the experimental group (*n* = 32) were applied the open‐heart surgery patient care protocol, and those in the control group (*n* = 32) were given the existing nursing care of the intensive care. The findings obtained from the study were discussed under the following headings in line with the literature.

### 7.1 Discussion of findings about the effect of the protocol on pain levels

Patients in both groups experienced mild pain; however, the pain score in the experimental group was found to be significantly lower because of the effect of the protocol. Control group patients reported that the sternum area, back, saphenous vein incision and drain area were the most painful, respectively. Patients undergoing cardiac surgery feel pain in the sternum because of sternotomy and in the saphenous area because of saphenous vein incision.^40^ Sternal incisions, drain areas and saphenous incisions may hinder the movement of patients and cause respiratory system deterioration. For this reason, nurses can evaluate the pain areas of patients undergoing cardiac surgery and implement site‐specific applications. In addition, intensive care nurses should increase their awareness about pain‐related movement and activity limitations and plan appropriate nursing interventions. It was noteworthy that the second most common area of pain in the control group patients in the study was the back. On the other hand, the region with the least pain was the back in the experimental group. It is thought that the difference may have been because of a practice in the care protocol, which included changing the bed position as the patient wished or every 2 h.

It was determined in the study that the protocol reduced the pain of patients with high pain levels. Non‐pharmacological nursing practices regarding pain management in patients undergoing cardiac surgery have been examined in the literature,^41^ but there is no study on the examination of the effect of a care protocol. A care protocol requires multidisciplinary team collaboration and longer follow‐up, which may make intensive care nurses' adjustment difficult. For this reason, studies aiming to develop and implement a protocol and monitor its results may not have been preferred.

It has been reported that anxiety affects postoperative pain and analgesic use (Arpag and Öztekin, 2023^42^, ^43^;). In a study by Arpag and Öztekin (2023),^44^ in which operating room nurses also participated in patient care, it was reported that this practice contributed to the anxiety and pain management and reduction of opioid use in patients undergoing cardiac surgery (Arpag and Öztekin, 2023). It has been shown that the level of anxiety experienced before surgery in patients undergoing similar surgery affects the level of pain and need for analgesics following surgery.^42^, ^43^ The use of multimodal or non‐opioid analgesics is recommended in the literature for patients undergoing cardiac surgery.^8^ It has been reported that opioid use during and after cardiac surgery causes side effects, such as respiratory depression, ileus, urinary retention, constipation, delirium, itching, nausea and vomiting, and also increases the risk for opioid addiction if the pain becomes chronic.^45^ Considering the serious side effects of opioids, it is thought that intensive care nurses' use of different nursing practices as the first choice in pain that develops following cardiac surgery may be possible by developing protocols such as the one in our study.

Mahjoubifard et al.^46^ reported that paracetamol was a good choice for reducing post‐CABG pain without significant complications compared with opioids.^46^ Additionally, in randomized controlled studies conducted with patients undergoing cardiac surgery, it was stated that average pain and opioid consumption decreased with the use of NSAIDs.^47^ In our study, it was revealed that the amount of analgesic use by the patients in the experimental group decreased throughout the intensive care period and they used fewer opioid‐type analgesics compared with the control group. Our study findings show that starting non‐pharmacological nursing interventions before pain occurs in patients undergoing open‐heart surgery may be effective in reducing opioid use. It is thought that it is important for intensive care nurses to primarily use non‐pharmacological methods and then gradually pharmacological approaches in the pain management of patients in intensive care following open‐heart surgery.

According to a study, surgical nurses do not provide their patients with enough information about postoperative pain.^48^ In our study, patients were informed about open‐heart surgery and pain management before surgery. This resulted in high levels of quality of care provided for patients in the experimental group for postoperative pain management. It is anticipated that the information given by intensive care nurses to patients undergoing cardiac surgery before and after the surgery will facilitate postoperative pain management.

### 7.2 Discussion of findings about the effect of the protocol on anxiety levels

In our study, it was found that patients experienced anxiety before the open‐heart surgery. Doğrusöz and Öztürk^17^ stated that patients experienced moderate anxiety before surgery.^17^ Albanesi et al.^49^ emphasized that nurses and nursing care interventions played a key role in reducing the anxiety experienced by patients in intensive care following cardiac surgery.^49^ Anxiety sensitivity occurring in intensive care can be shaped by evidence‐based interventions provided by health care professionals.^50^ In light of the findings, it was revealed that the anxiety levels of the experimental group patients who were applied the open‐heart surgery patient care protocol decreased.

No study has been found in the literature on the examination of the effect of the care protocol on the anxiety of patients undergoing open‐heart surgery. There are studies on the evaluation of the anxiety levels of patients undergoing cardiac surgery by applying different non‐pharmacological and supportive nursing interventions.^15^, ^17^, ^42^ It is envisaged that non‐pharmacological nursing interventions applied to patients who will undergo open‐heart surgery and covering the entire pre‐ and post‐surgical process are an important step in controlling the level of anxiety.

In a study by Chang and Tsai,^19^ patients undergoing cardiac surgery stated that they wanted to be informed about the postoperative process before the surgery and receive multidisciplinary care.^19^ In a similar study, it was reported that supportive educational interventions accompanied by a nurse before CABG surgery reduced the anxiety levels of patients.^20^ According to the results in the literature, education and information provided for patients by intensive care nurses before surgery may reduce the level of anxiety experienced after surgery. Care covers a period from patient admission to discharge. It is thought that the better intensive care nurses can manage this process holistically, the more anxiety and the symptoms it causes can be controlled.

One of the most important factors that cause anxiety after surgery is the experience of pain.^16^ According to a study, there is a relationship between anxiety before cardiac surgery and pain experienced following surgery, and patients experience anxiety before surgery.^51^ There is a cyclical relationship between pain and anxiety that cause each other.^16^ In particular, it is one of the human rights of patients to have their anxiety managed by their primary care nurses.^16^ It has also been stated that not catastrophizing pain by patients and nurses will reduce anxiety and increase the quality of pain management.^51^ Dağcan et al.^52^ reported that the pain experienced after cardiac surgery negatively affected patients and increased their anxiety levels by causing distress and helplessness.^52^

Cardiac surgery alone can be a source of anxiety. Because of the meaning of life attributed to the heart, heart surgeries are associated with death by individuals. This causes the situation to be frightening and leads to high anxiety.^1^ In this study, while the initial anxiety of the patients in both groups was high, the final anxiety measurement results were positively affected because their expectations regarding the success of the surgery were realized and their pain decreased.

### 7.3 Discussion of the findings about the effect of the protocol on the quality of care given to the patient for post‐operative pain management

Some studies have shown that good relationships between patients and nurses^53^ and adequate knowledge and skill levels of nurses increase the quality of care.^22^ In our study, the high care scores of the patients in the experimental group indicated that they were satisfied with the intensive care nursing interventions for pain and anxiety. The results might indicate that the intensive care nursing interventions applied according to the protocol for pain and anxiety were implemented correctly and completely by the nurses and that they positively affected the quality of care by increasing the communication between the patient and the nurse.

There are no studies in the literature showing the effects of an intensive care nursing care protocol on the level of quality of care, but there are studies on the satisfaction of patients with the quality of nursing care following surgery.^54^ In our study, the management of pain with a gradual and continuous care protocol, as recommended by the guidelines, and the delivery of the protocol by trained and experienced specialist intensive care nurses may have caused the quality of care to be perceived as high. It is thought that the open‐heart surgery patient care protocol positively affected the quality of patient care.

There are studies in the literature on the examination of pain levels and satisfaction with the quality of care. As the level of pain decreases, the level of satisfaction with the quality of care increases.^55^ In our study, it was determined that the experimental group patients were satisfied with the quality of care provided. After all, there are results in the literature obtained from different sample groups regarding post‐surgical pain and the quality of intensive care nursing care, but it is thought that pain and care quality satisfaction are interrelated concepts. It is considered that intensive care nurses' preference for especially non‐pharmacological interventions for pain relief after open‐heart surgery will affect the quality of care given to the patient.

## 8 LIMITATIONS

The protocol was developed only for the cardiac surgical intensive care unit and was evaluated on intensive care patients. After the patients were transferred from the intensive care unit to the clinic, it was not followed whether the effects of the protocol on pain continued. Another limitation was that the time the patients spent after surgery until they were extubated was not included in the study.

## 9 RECOMMENDATIONS OR IMPLICATIONS FOR PRACTICE AND/OR FURTHER RESEARCH

It may be recommended to use the protocol in different groups of patients undergoing cardiac surgery, to plan longitudinal studies to determine the continuity of its effect and to conduct studies to evaluate the experiences of the intensive care nurses involved in the protocol and the patients to whom the protocol has been applied.

## 10 CONCLUSION

In this study, which was conducted to develop an open‐heart surgery patient care protocol and examine its effects on post‐sternotomy pain, the protocol reduced the post‐sternotomy pain, the use of NSAIDs and opioids and the level of anxiety developing following surgery, and increased the quality of care given to the patient for postoperative pain management. The protocol was original and feasible in that it included independent critical care nursing interventions to improve quality of care by reducing pain and anxiety.

## AUTHOR CONTRIBUTIONS

(1) Conception and design of the study, or acquisition of data, or analysis and interpretation of data: NDS and GGA; (2) drafting the article or revising: NDS and GGA; (3) final approval of the version to be submitted: NDS and GGA.

## CONFLICT OF INTEREST STATEMENT

The authors declared no potential conflicts of interest with respect to the research, authorship, and/or publication of this article.

## ETHICS STATEMENT

The patients were informed about the research and their written and verbal consent was obtained. The permission of the institution where the research would be conducted (05.08.2021), the approval of the Non‐Interventional Research Ethics Committee (decision number: 2021/17‐11; date: 02.06.2021), and the written permission of Vatansever and Akansel for the ‘Strategic and Clinical Quality Indicators in Postoperative Pain Management Questionnaire’ used in the research were obtained. The study was registered at ClinicalTrials (registration code: NCT05902052).

## Data Availability

The data that support the findings of this study are available on request from the corresponding author. The data are not publicly available due to privacy or ethical restrictions.

## References

[1] Dağcan Şahin N , Gürol Arslan G , Özbek D . Factors affecting death anxiety in patients undergoing open heart surgery: a cross‐sectional study. Omega (Westport). 2023;1‐13. doi:10.1177/00302228231214128 37933629

[2] World Health Organization (WHO) . Cardiovascular diseases (CVDs). 2021 Accessed October 06, 2023. https://www.who.int/en/news-room/fact-sheets/detail/cardiovascular-diseases-(cvds)

[3] Dagcan N , Özden D , Gürol AG . Nursing care of individual undergoing open hearth surgery according to the ROY adaptation model, NANDA, NIC and NOC classification systems. Turkiye Klinikleri J Nurs Sci. 2021;13(4):1019‐1028. doi:10.5336/nurses.2020-80569

[4] Seo Y , Lee HJ , Ha EJ , Ha TS . 2021 KSCCM clinical practice guidelines for pain, agitation, delirium, immobility, and sleep disturbance in the intensive care unit. Acute Crit Care. 2022;37(1):1‐25. doi:10.4266/acc.2022.00094 35279975 PMC8918705

[5] Bordoni B , Marelli F , Morabito B , Sacconi B , Severino P . Post‐sternotomy pain syndrome following cardiac surgery: case report. J Pain Res. 2017;10:1163‐1169. doi:10.2147/JPR.S129394 28553137 PMC5439996

[6] Taherian T , Shorofi SA , Zeydi AE , Charati JY , Pouresmail Z , Jafari H . The effects of Hegu point ice massage on post‐sternotomy pain in patients undergoing coronary artery bypass grafting: a single–blind, randomized, clinical trial. Adv Integr Med. 2020;7(2):73‐78. doi:10.1016/j.aimed.2019.08.001

[7] Balas MC , Weinhouse GL , Denehy L , et al. Interpreting and implementing the 2018 pain, agitation/sedation, delirium, immobility, and sleep disruption clinical practice guideline. Crit Care Med. 2018;46(9):1464‐1470. doi:10.1097/CCM.0000000000003307 30024427

[8] Grant MC , Chappell D , Gan TJ , et al. Pain management and opioid stewardship in adult cardiac surgery: joint consensus report of the PeriOperative Quality Initiative and the Enhanced Recovery after surgery cardiac society. J Thorac Cardiovasc Surg. 2023;166(6):1695‐1706.e2. doi:10.1016/j.jtcvs.2023.01.020 36868931

[9] Hyland SJ , Wetshtein AM , Grable SJ , Jackson MP . Acute pain management pearls: a focused review for the hospital clinician. Healthcare (Basel). 2022;11(1):34. doi:10.3390/healthcare11010034 36611494 PMC9818465

[10] Devlin JW , Skrobik Y , Gélinas C , et al. Clinical practice guidelines for the prevention and management of pain, agitation/ sedation, delirium, immobility, and sleep disruption in adult patients in the ICU. Crit Care Med. 2018a;46(9):e825‐e873. doi:10.1097/CCM.0000000000003299 30113379

[11] Devlin JW , Skrobik Y , Rochwerg B , et al. Methodologic innovation in creating clinical practice guidelines: insights from the 2018 society of critical care medicine pain, agitation/sedation, delirium, immobility, and sleep disruption guideline effort. Crit Care Med. 2018b;46(9):1457‐1463. doi:10.1097/CCM.0000000000003298 29985807

[12] Chou R , Gordon DB , Leon‐Casasola OA , et al. Management of postoperative pain: a clinical practice guideline from the american pain society, the american society of regional anesthesia and pain medicine, and the american society of anesthesiologists' committee on regional anesthesia, executive committee, and administrative council. J Pain. 2016;17(2):131‐157. doi:10.1016/j.jpain.2015.12.008 26827847

[13] Yıldırım M , Akbal S , Turkoglu M . The effect of self‐affirmation on anxiety and perceived discomfort in patients who have undergone open‐heart surgery. A randomized controlled trial. Appl Nurs Res. 2023;72:151687. doi:10.1016/j.apnr.2023.151687 37423676

[14] Seifi Z , Beikmoradi A , Oshvandi K , Poorolajal J , Araghchian M , Safiaryan R . The effect of lavender essential oil on anxiety level in patients undergoing coronary artery bypass graft surgery: a double‐blinded randomized clinical trial. Iran J Nurs Midwifery Res. 2014;19(6):574‐580. 25558253 PMC4280720

[15] Göktuna G , Dağcan N , Arslan GG . The effect of hand reflexology massage on pain and anxiety after coronary artery bypass graft surgery: a double‐blind, randomized, placebo‐controlled trial. J Cardiovasc Nurs. 2023. doi:10.1097/JCN.0000000000001033 37548394

[16] Tat Çatal A , Cebeci F . Pain, anxiety, depression cycle and the role of nurses in lumbar disc herniation. J Hacet Univ Fac Nurs. 2020;7(1):73‐77.

[17] Doğrusöz P , Öztürk Ş . The effect of music on anxiety and pain level in patients who had coronary artery bypass graft surgery. Fenerbahçe Univ J Health Sci. 2023;3(1):78‐91. doi:10.56061/fbujohs.1163208

[18] Abdelhakim AM , Hussein AS , Doheim MF , Sayed AK . The effect of inhalation aromatherapy in patients undergoing cardiac surgery: a systematic review and meta‐analysis of randomized controlled trials. Complement Ther Med. 2020;48:102256. doi:10.1016/j.ctim.2019.102256 31987220

[19] Chang YL , Tsai YF . Early illness experiences related to unexpected heart surgery: a qualitative descriptive study. Aust Crit Care. 2017;30(5):279‐285. doi:10.1016/j.aucc.2016.11.005 28063723

[20] Mousavi Malek N , Zakerimoghadam M , Esmaeili M , Kazemnejad A . Effects of nurse‐led intervention on patients' anxiety and sleep before coronary artery bypass grafting. Crit Care Nurs Q. 2018;41(2):161‐169. doi:10.1097/CNQ.0000000000000195 29494371

[21] Uzelli Yılmaz D , Akın Korhan E , Khorshid L . Evulation of nursing care quality in a palliative care clinic. J Hum Sci. 2017;14(3):2968‐2980. doi:10.14687/jhs.v14i3.4828

[22] Gülen D , Zaybak A . The perception of nursing care quality by patients and nurses: descprictive study. Turkiye Klinikleri J Nurs Sci. 2023;15(1):108‐117. doi:10.5336/nurses.2022-89802

[23] Kızılcık Özkan Z , Dığın F , Dinlegör SI . Perioperative nursing care quality in patients and affecting factors. Celal Bayar Univ Health Sci Inst J. 2023;10(1):26‐32. doi:10.34087/cbusbed.1147163

[24] Reisli R , Akkaya ÖT , Arıcan Ş , et al. Pharmachologic treatment of acute postoperative pain: a clinical practice guideline of the Turkish Society of Algology. Ağrı. 2021;33(1):1‐51. doi:10.14744/agri.2021.60243 33523457

[25] Schulz KF , Altman DG , Moher D , CONSORT Group . CONSORT 2010 statement: updated guidelines for reporting parallel group randomised trials. BMC Med. 2010;8:18. doi:10.1186/1741-7015-8-18 20619135

[26] Chandrababu R , Nayak BS , Pai VB , et al. Effects of foot massage and patient education in patients undergoing coronary artery bypass graft surgery: a randomized controlled trial. Complement Ther Clin Pract. 2020;40:101215. doi:10.1016/j.ctcp.2020.101215 32891291

[27] Cohen J . Statistical Power Analysis for the Behavioral Sciences. 2nd ed. Routledge; 1988.

[28] Faul F , Erdfelder E , Lang AG , Buchner A . G*power 3: a flexible statistical power analysis program for the social, behavioral, and biomedical sciences. Behav Res. 2007;39:175‐191. doi:10.3758/BF03193146 17695343

[29] Bijur PE , Latimer CT , Gallagher EJ . Validation of a verbally administered numerical rating scale of acute pain for use in the emergency department. Acad Emerg Med. 2003;10(4):390‐392. doi:10.1111/j.1553-2712.2003.tb01355.x 12670856

[30] Karcioglu O , Topacoglu H , Dikme O , Dikme O . A systematic review of the pain scales in adults: which to use? Am J Emerg Med. 2018;36(4):707‐714. doi:10.1016/j.ajem.2018.01.008 29321111

[31] Spielberger CD . State‐Trait Anxiety Inventory: Bibliography. 2nd ed. Consulting Psychologists Press; 1989.

[32] Öner N , Compte A . State‐Trait Anxiety Inventory Manual. Boğaziçi University Printing House; 1985.

[33] Idvall E , Hamrin E , Unosson M . Development of an instrument to measure strategic and clinical quality indicators in postoperative pain management. J Adv Nurs. 2002;37(6):532‐540. doi:10.1046/j.1365-2648.2002.02130.x 11879417

[34] Vatansever NA , Akansel N . Validation study of the strategic and clinical quality indicators in postoperative pain management questionnaire in Turkish surgery patients. Pain Manag Nurs. 2014;15(4):871‐880. doi:10.1016/j.pmn.2014.01.003 24981119

[35] Özçelik H , Fadıloğlu Ç , Karabulut B v a . Case management based multidisciplinary care protocol in the palliative care of cancer patients. J Turkish Soc Algol. 2014;26(2):47‐56. doi:10.5505/agri.2014.93585 24943853

[36] Tura İ , Erden S . Evidence‐based recommendations for postoperative pain control. Dent Med J‐R. 2022;4(1):34‐47.

[37] Van Gulik L , Ahlers SJ , Bruins P , Tibboel D , Knibbe CA , van Dijk M . Adherence to all steps of a pain management protocol in intensive care patients after cardiac surgery is hard to achieve. Pain Res Manag. 2017;2017:7187232. doi:10.1155/2017/7187232 28298879 PMC5337384

[38] Davis LL . Instrument review: getting the most from a panel of experts. Appl Nurs Res. 1992;5:194‐197. doi:10.1016/S0897-1897(05)80008-4

[39] Smith GB , Recio‐Saucedo A , Griffiths P . The measurement frequency and completeness of vital signs in general hospital wards: an evidence free zone. Int J Nurs Stud. 2017;74:A1‐A4. doi:10.1016/j.ijnurstu.2017.07.001 28701265

[40] Seyhan AE , Ayoğlu T , Kandemi̇r D . New approaches in pain control following cardiac surgery. Izmir Katip Celebi Univ Faculty Health Sci J. 2017;2(1):29‐32.

[41] Liu M , Ni R , Huang S , et al. Efficacy of non‐pharmacological interventions in pain relief and opioid consumption after cardiac surgery: a systematic review and Bayesian network meta‐analysis. J Clin Nurs. 2023;32(15–16):4626‐4637. doi:10.1111/jocn.16482 35949177

[42] Ertürk EB , Ünlü H . Effects of pre‐operative individualized education on anxiety and pain severity in patients following open‐heart surgery. Int J Health Sci. 2018;12(4):26. PMC6040857 30022900

[43] Kalogianni A , Almpani P , Vastardis L , Baltopoulos G , Charitos C , Brokalaki H . Can nurse‐led preoperative education reduce anxiety and postoperative complications of patients undergoing cardiac surgery. Eur J Cardiovasc Nurs. 2016;15(6):447‐458. doi:10.1177/1474515115602678 26304701

[44] Arpag N , Öztekin SD . The effect of visits by operating room nurses before cardiac surgery on anxiety and pain management. J Perianesth Nurs. 2023;38(6):892‐900. doi:10.1016/j.jopan.2023.01.022 37330723

[45] Krakowski JC , Hallman MJ , Smeltz AM . Persistent pain after cardiac surgery: prevention and management. Semin Cardiothorac Vasc Anesth. 2021;25(4):289‐300. doi:10.1177/10892532211041320 34416847 PMC8669213

[46] Mahjoubifard M , Moeini Y , Feizabad E , Abdolrazaghnejad A . The effect of paracetamol on the patient‐controlled pain after coronary artery bypass surgery: a randomized clinical trial study. J Pharm Care. 2021;9(4):176‐182. doi:10.18502/jpc.v9i4.8222

[47] Qazi SM , Sindby EJ , Nørgaard MA . Ibuprofen a safe analgesic during cardiac surgery recovery? A randomized controlled trial. J Cardiovasc Thorac Res. 2015;7:141‐148. doi:10.15171/jcvtr.2015.31 26702342 PMC4685279

[48] Kapikiran G , Güneş H , Bülbüloğlu S , Saritas S . The level of Patients' satisfaction with practices related to pain after abdominal surgery: a descriptive study. Turkiye Klinikleri J Nurs Sci. 2021;13(4):786‐794. doi:10.5336/nurses.2021-82032

[49] Albanesi B , Nania T , Barello S , et al. Lived experience of patients in ICU after cardiac surgery: a phenomenological study. Nurs Crit Care. 2022;27(2):204‐213. doi:10.1111/nicc.12562 33063374

[50] Boehm LM , Bird CM , Warren AM , et al. Understanding and managing anxiety sensitivity during critical illness and long‐term recovery. Am J Crit Care. 2023;32(6):449‐457. doi:10.4037/ajcc2023975 37907373 PMC10718181

[51] Tai AL , Hsieh HF , Chou PL , Chen HM , Liu Y . The influence of preoperative anxiety, optimism, and pain catastrophizing on acute postoperative pain in patients undergoing cardiac surgery: a cross‐sectional study. J Cardiovasc Nurs. 2021;36(5):454‐460. doi:10.1097/JCN.0000000000000687 32501863

[52] Dağcan N , Özden D , Gürol AG . Pain perception of patients in intensive care unit after cardiac surgery: A qualitative study using Roy's Adaptation Model. Nurs Crit Care. 2023;28(5):1‐9. doi:10.1111/nicc.12958 37527978

[53] Olowe AFF , Odeyemi O . Assessment of patient satisfaction with nursing care in selected wards of the Lagos university teaching hospital (luth). Biomed J Sci Tech Res. 2019;17(1):12489‐12497. doi:10.26717/BJSTR.2019.17.002941

[54] Yıldız Fındık Ü , Soydaş YD . Surgical patients' perception of the postoperative nursing care quality. J Anatolia Nurs Health Sci. 2017;20(3):195‐200.

[55] Eti Aslan F , Kula Şahin S , Secginli S , Bülbüloğlu S . Patient satisfaction with nursing practices about postoperative pain management: a systematic review. J Turkish Soc Algol. 2018;30(3):105‐115. doi:10.5505/agri.2018.96720 30028476

